# Cardiovascular adverse effects associated with the use of anti-HER2 in breast cancer treatment

**DOI:** 10.3389/fphar.2023.1099545

**Published:** 2023-09-18

**Authors:** Patricia Marques Soares Valente, Paula Nogueira da Silva, Licínio Esmeraldo da Silva, Wolney de Andrade Martins, Selma Rodrigues de Castilho

**Affiliations:** ^1^ Programa de Pós-Graduação em Ciências Aplicadas à Produtos para a Saúde, Faculdade de Farmácia, Universidade Federal Fluminense, Niterói, Brazil; ^2^ Grupo Oncoclínicas, Rio de Janeiro, Brazil; ^3^ Departamento de Estatística, Faculdade de Matemática, Universidade Federal Fluminense, Niterói, Brazil; ^4^ Curso de Pós-Graduação em Ciências Cardiovasculares, Faculdade de Medicina, Universidade Federal Fluminense, Niterói, Brazil

**Keywords:** monoclonal antibodies, receptor HER-2, cardiovascular adverse events, breast cancer, pharmacoepidemiology

## Abstract

**Background:** Cancer represents an important public health problem with increasing incidence, prevalence, and mortality, affecting the entire Western population, especially in developed and developing countries. The use of monoclonal antibodies has revolutionized the treatment of cancer, but this treatment can cause adverse cardiovascular effects (AE).

**Objective:** The objective of this paper is to identify and classify AE in breast cancer patients in the use of Trastuzumab in two health institutions.

**Methods:** Retrospective study of medical records of patients with breast cancer Her 2+ submitted the therapy with trastuzumab in early and advanced stage of the disease. Review conducted in a university hospital and a private clinic, both located in Rio de Janeiro State, Brazil.

**Results:** Cardiovascular events were late for trastuzumab, with predominance of moderate reactions. There was a predominance of dyspnea, increased blood pressure, fatigue and reduced left ventricular ejection.

**Conclusion:** The results resemble similarities in the pattern of the institutions’ reactions. Identify possible AE and know the toxicity profile of trastuzumab can contribute to a safer therapy.

## Introduction

Cancer is a significant public health problem worldwide and is among most countries’ leading causes of death before age 70. Studies indicate that the incidence and mortality rate from cancer has been increasing worldwide due to population aging, as well as associated with the change in the distribution and prevalence of cancer risk factors, especially those associated with the socioeconomic development of the population. There is a transition in the main types of cancer observed in developing countries, with a decline in types of cancer associated with infections and an increase in those associated with the improvement of socioeconomic conditions with the incorporation of habits and actions associated with the urbanization of countries (Bray, et al., 2018).

World data indicate breast cancer is the primary global incidence cause, with 11.7% of the total cases. In 2020, about 2.3 million new cases, equivalent to 24.5% of all cancers in women, excluded non-melanoma skin. This value corresponds to the estimated risk of 47.80 cases per 100,000 women. The highest estimated incidence rates were in North America, Western Europe, and Oceania (Ferlay and Sung et al., 2021). Cancer treatment has advanced a lot in recent decades, allowing for more remarkable survival and increased life expectancy of survivors. Monoclonal antibodies anti-HER-2 constitute an essential group of drugs obtained by biotechnology, contributing to this improvement. Her-2 belongs to a family of four transmembranes that receive tyrosine kinases involved in growth, differencing, and cellular survival. Using monoclonal antibodies made it possible to reduce common adverse events related to standard chemotherapy because they selectively act on cancer cells (Chan and Hughes, 2015; Pérez- Herrero e Fernández -Medard, 2015; Piccart-Gebhart, M. et al., 2005). Trastuzumab is a monoclonal antibody that acts on the epidermal growth factor 2. Although this receptor plays an essential role in the normal growth and development of various cell types, its overexpression in 20%–25% of breast cancer is associated with a worse prognosis.

So Trastuzumab became the first “MAB” used clinically against HER-2. It was described primarily with prognostic relevance in breast cancer by Slamon et al., (1987).

The Food and Drug Administration (FDA) approved Trastuzumab in 1998. Still, in trastuzumab phase III tests, the first cases of cardiotoxicity in patients were reported, and its effect was first attributed to the prior use of anthracyclines (Seidman et al., 2002; Nemeth et al., 2017). However, they are relatively new drugs in use by the Brazilian Unified Health System, requiring more information about adverse reactions during and after using these drugs. Phase 3 studies of Trastuzumab used in patients with metastatic HER2+ breast cancer indicated the occurrence of serious AE such as cardiac dysfunction, dyspnea, asthenia, leukopenia, and infusion-related reactions (Piccart-Gebhart, M. et al., 2005).

The present study aims to report the main cardiovascular AE associated with using the monoclonal antibody trastuzumab in breast cancer patients in a university hospital and a private oncology clinic, presenting their similarities and differences.

## Methodology

### Study design

A retrospective study of medical records review was conducted in a university hospital and a private clinic in Rio de Janeiro State, Brazil. The university hospital serves adult cardiology, hematology, and oncology patients. The private clinic is specialized in oncology and serves adult and pediatric patients in onco-hematological treatment.

### Development

Patient selection: Adult patients with HER-2-positive breast cancer who used Trastuzumab.

Study period: For the university hospital, the selected study period was from 2013 to 2018. The selected period is in accordance with the approval of Trastuzumab for use in public hospitals by the National Commission for the Incorporation of Technologies (CONITEC) in 2015.

For the oncology clinic, the selected period was from 2018 to 2020.

Inclusion criteria: patients over 18 years of age and with HER-2 positive breast cancer on adjuvant and palliative trastuzumab treatment.

Exclusion criteria: patients with other neoplasms or incomplete medical records.

The project was approved by the Research Ethics Committee of the university hospital and the private clinic under the numbers: 98429018000005243 and 38594120.9.0000.5243, respectively.

### Data collection

Data were collected from physical and digital medical records, using a form adapted and previously validated by the cardio-oncology study group of the university hospital. The adaptation involved the inclusion of the Naranjo Algorithm (Naranjo et al., 1981; Sobrafo, 2011) and the algorithm formulated by the Mayo Clinic (Barros Gomes et al., 2016) for cardiovascular risk stratification. Cardiovascular AE were classified into definite, probable, possible, and doubtful, and the Mayo Clinic risk score was classified into extreme, high, intermediate, low, and very low risk. Information was collected based on variables: age, gender, obesity (calculated by the body mass index), smoking, alcohol consumption, previous use of anthracyclines, radiotherapy, and presence of previous cardiovascular diseases (hypertension, diabetes mellitus, and dyslipidemia).

### Cardiotoxicity criteria

The cardiotoxicity assessed in the study was heart failure, defined by the American Society of Echocardiography and the European Association of Cardiovascular Imaging for cardiotoxicity studies, which represents a reduction in LVEF below 53% or a 10% reduction from baseline, with or without symptoms, and repeating the exam 2–3 weeks later (Plana et al., 2014; Lyon et al., 2022).

### Data analysis

Data were analyzed using descriptive statistics tools. Continuous variables were expressed as means and standard deviation. Categorical variables were expressed as absolute numbers or percentages. The chi-square test was used to assess the associations of variables with the occurrence of CI, with a significance level of 95%. Odds ratios (OR) and confidence intervals [CI] were calculated at 95%. All statistical analyzes were performed using PASW Statistics for Windows, Version 18.0 (SPSS Inc, Chicago, IL).

## Results

### Unit A-university hospital

**FIGURE 1:**
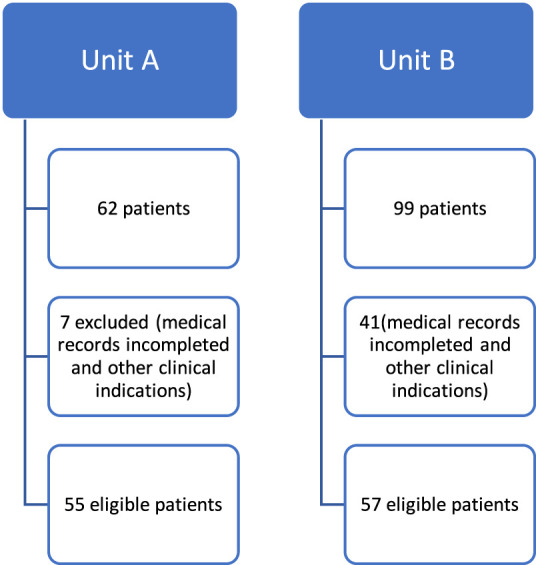
Flowchart of patients undergoing trastuzumab therapy in two health institutions (Unit A and Unit B).

The study population comprised 55 patients undergoing treatment with the monoclonal antibodies Trastuzumab (Figure 1). The sample population was exclusively female, with a median age of and predominance of Infiltrating Ductal Carcinoma (IDC) (100%) (Table 1). Hypertension was the most prevalent (53%) comorbidity found in the study, followed by smoking (33%), obesity (29%), diabetes mellitus (22%), alcohol consumption (9%), dyslipidemia (6%) and sedentary lifestyle (4%). In the analyzed group, 47% used anthracyclines (Table 1).

**TABLE 1:** Baseline characteristics of patients.

Risk factors	Unit A, *n* = 55 n (%)	Unit B, *n* = 57 n (%)
Hypertension	29 (53)	26 (46)
Diabetes	12 (22)	11 (19)
Obesity	16 (29)	14 (24)
Dyslipidemia	3 (6)	13 (23)
Sedentary lifestyle	3 (4)	47 (82)
Smoking	18 (33)	15 (26)
Alcohol consumption	5 (9)	31 (54)
Anthracycline therapy	26 (47)	30 (53)
Radiotherapy	31 (56)	50 (88)

Cardiovascular reactions were identified in 18 patients, mainly during infusion and in the early phase. Most reactions were classified as probable (67%) and were classified as moderate in severity.

The main reactions were dyspnea (13%), increased blood pressure (13%), fatigue (11%), and arrhythmias (9%) (Table 2). Among the analyzed patients, 4 (8%) had reduced left ventricular ejection fraction (LVEF) or signs and symptoms of heart failure. The pharmacological groups most used to treat hypertension were angiotensin receptors antagonists (26%), angiotensin converter enzyme inhibitors (20%), diuretics thiazide (18%), selective beta-blockers (15%), calcium channel antagonists (4%), and others (Table 2).

**TABLE 2:** Symptoms and signs of cardiotoxicity.

Symptoms/Signs	Unit A, *n* = 55 n (%)	Unit B, *n* = 57 n (%)
Dyspnea	7 (13)	1 (2)
Hypertensive peak	7 (13)	9 (16)
Fatigue	6 (11)	43 (75)
Arrhthmia	5 (9)	1 (2)
Weight gain	4 (7)	-
Hipotension	-	10 (18)
Weight low	-	10 (18)
Night cough	-	2 (35)

Regarding the cardiovascular risk score assessment proposed by the Mayo Clinic, there was a higher prevalence of extreme-risk patients (91%) and high-risk patients (9%).

### Cardiotoxicity in patients

Twenty-eight patients (50.9%) had some cardiovascular disorder during or after cancer treatment. Of the 28 patients who had cardiovascular events, seven (12.7%) had infusion-related cardiovascular effects; twelve (21.9%) developed HF; and nine (16.4%) developed comorbidities during or after treatment, such as hypertension, diabetes mellitus, dyslipidemia, weight gain, and metabolic syndrome. Of the 12 patients who developed the HF group, five had a decline in LVEF below 53%, four with signs and symptoms without a decline in LVEF, and three patients had a decline greater than 10% from baseline.

### Statistics analysis

Among the analyzed variables, only for smoking, the odds ratio showed an increased risk for the occurrence of heart failure (OR = 17.578 CI (OR: 95%): [1.998; 154.661], although the wide confidence interval indicates that caution is needed in the analysis. The values for cardiovascular disease (CAD), cerebrovascular accident (CVA), dyslipidemia, and sedentary lifestyle were shallow, and it was impossible to calculate the *p*-value.

The values are shown in Table 1. The chi-square test was used to assess the association between risk factors and the development of heart failure, noting that only smoking had a significant result (*p* = 0.0325), considering *p* < 0.05. The values of CAD, CVA, dyslipidemia, and sedentary lifestyle were shallow, and it was impossible to calculate the *p*-value.

The chi-square test values are shown in Table 3.

**TABLE 3:** Risk factors evaluated for association with trastuzumab cardiotoxicity.

Risk factors	Group with cardiotoxicity Unit A *n* = 55 n (%)	Group with cardiotoxicity Unit B *n* = 57 n (%)	Group without cardiotoxicity Unit A *n* = 55 n (%)	Group without cardiotoxicity Unit B *n* = 57 n (%)	*P-value* Unit A	*P-value* Unit B
Patients	12 (22)	12 (21)	43 (78)	45 (79)	-	-
Hypertension	29 (53)	26 (46)	24 (56)	18 (40)	0,39	0,09
Diabetes	12 (22)	11 (19)	10 (23)	6 (13)	0,63	0,02
Obesity	16 (29)	14 (24)	13 (30)	12 (27)	0,72	0,47
Smoking	18 (33)	15 (26)	11 (26)	11 (24)	0,03	0,53
Alcohol consumption	5 (9)	31 (54)	5 (12)	22 (49)	0,75	0,10
Anthracycline therapy	26 (47)	30 (53)	25 (58)	21 (47)	0,31	0,08
Radiotherapy	31 (56)	50 (88)	26 (61)	40 (89)	0,52	0,60

### Unit B-private oncology clinic

The study population of Unit B comprised patients undergoing treatment with Trastuzumab and included 57 patients (Figure 1), with a median age of 54 years and a range of 27–77 years. All patients were female; the most prevalent histological type was IDC (91%). The majority (72%) of patients were receptor positive for hormone therapy, and most were treated with anthracyclines (52%), including doxorubicin or epirubicin, with monitoring of LVEF and symptoms suggestive of cardiovascular adverse reactions if necessary (Table 1).

Regarding the twelve patients who had adverse cardiovascular reactions, the following previous comorbidities stood out: hypertension (58%), previous heart disease (50%), dyslipidemia (42%), and diabetes mellitus (42%). Patients mainly used drugs: enalapril, hydrochlorothiazide, valsartan, and losartan. The most prominent risk factors in the study were: exposure to radiotherapy in all 12 patients, Use of anthracyclines by 75%, and age> 60 years in 42%. Regarding modifiable risk factors, alcohol consumption was present at 83% and smoking at 33%. Both were the most prevalent. The suggestive symptoms of cardiovascular reactions highlighted in the study were fatigue and weight loss (Table 2).

### Cardiotoxicity in patients

Twelve patients (21%) had late AE, such as left ventricular dysfunction and reduced LVEF, requiring antibody suspension or interruption. Regarding these 12 patients, 67% had an extreme or high-risk score in the cardiotoxicity risk assessment algorithm. Regarding Naranjo’s causality, eight patients had probable results and four as possible AE.

### Statistics analysis

Only diabetes mellitus (*p* = 0.02) showed a significant difference as prior comorbidity associated with increased risk of cardiotoxicity development (Table 3). AE was observed in 12 patients (21%), including respiratory discomfort, rash, headache, and hyperemia.

## Discussion

For unit A, most cardiovascular reactions were infusion, early, possible, and grade 2 (moderate). Bruneto et al. found similar results (Bruneto et al., 2019). The main cardiovascular reactions were dyspnea, increased blood pressure, and fatigue. Similar data were found in the study by Gomes da Fonseca et al. (2014) conducted in a Brazilian hospital, where the median age was 53 years old [27-83]. About 30.5% of patients had hypertension, diabetes (8.8%), and smoking (24.5%) (Gomes da Fonseca et al., 2014).

Hypertensive patients in the study used antihypertensives before cancer treatment, which may have protected the heart. Studies suggest cardiac protection for patients using antihypertensive classes such as losartan and enalapril, corroborating low heart toxicity indices in hypertensive patients. (Losartan and enalapril) (Guglin et al., 2009), Like in other studies, smoking was high (Gomes da Fonseca et al., 2014; Java et al., 2016) and showed a significant association between smoking with HF. For the other risk factors, there was no statistically significant association considering *p*˂0.05, which may have occurred due to this study’s small sample size (N = 55).

The analysis of cardiotoxicity, according to the LVEF criteria, identified that 21.8% of breast cancer patients treated with Trastuzumab developed HF, which is consistent with that found by Gomes da Fonseca et al., 2014, who found a value of 20.2% in a similar study using same cardiotoxicity criteria and design. These authors observed similar results for median age, BMI, hypertension, diabetes, smoking, radiotherapy, and use of anthracyclines (Gomes da Fonseca et al., 2014). Other studies conducted in other hospitals found higher CI values ranging from 28% to 32% (Ayres et al., 2015).

Guarneri et al., 2006; Onitilo et al., 2014; Vicente et al., 2007). For unit B, a similar result was observed in which the cardiovascular reactions were mainly: fatigue and weight loss. Those are common events associated with the use of Trastuzumab. Fatigue is also prevalent in 80%–90% of cancer patients treated with chemotherapy or radiotherapy and affects about 50%–96% of patients with heart failure (Evans et al., 2007; Borges et al., 2018).

Cardiovascular risk factors such as hypertension, diabetes mellitus, obesity, and smoking were prevalent in both units. These data are like the Brazilian epidemiological profile and like the study by Silva (2016), conducted in unit A and like the result of the Telephone Surveillance Study (VIGITEL) (Silva, 2016; Vigitel, 2019).

Hypertension represents the most frequently observed comorbidity in cancer patients, and its incidence tends to increase after cancer treatment. In both units, hypertension was present in most patients analyzed, corroborating other studies (Suter and Ewer, 2013; Souza et al., 2015).

Hypertension is a prevalent risk factor in the Brazilian population (Vigitel, 2019). Hypertension and cancer share risk factors such as obesity, a sedentary lifestyle, smoking, and alcohol consumption. On the other hand, the advance in developing new drugs for cancer treatment increased patient survival and contributed to the higher incidence of hypertension (De Souza et al., 2015).

Cancer therapy can aggravate hypertension, especially the VGEF inhibitors, such as bevacizumab. Controversially, the development of hypertension is associated with a better response to cancer treatment (Suter and Ewer, 2013; Souza et al., 2015). This relationship has been investigated in clinical trials that have sought to understand better this relationship known as “efficacy biomarkers.” The basis for this correlation may be pharmacological, with greater drug exposure associated with more significant toxicity and antitumor activity. However, it may also be genetic because single nucleotide polymorphisms are essential in pharmacokinetic and pharmacodynamic processes (Dienstmann et al., 2011).

Prior hypertension was the most prevalent risk factor in breast cancer, and the main medications used for prior hypertension included angiotensin receptor blockers (ARB), angiotensin-converting enzyme inhibitors (ACEI), thiazide diuretics and beta-blockers (BB). BRA, ACE inhibitors, and BB are groups of drugs that act as cardioprotectors, and it is recommended by the Cardio-Oncology Guideline 2020 that these drugs be considered for patients at higher risk of cardiotoxicity in the prevention of ventricular dysfunction and adverse cardiovascular events (Hajjar et al. al, 2020).

Although hypertension was prevalent, there was no significant association with AE. One explanation is that patients’ ARB, ACE inhibitors, and BB use contributed to the cardioprotective effect. A similar situation was observed in other studies (Gomes da Fonseca et al., 2014; Guglin et al., 2009).

Cardiovascular diseases in cancer patients are increasingly frequent events due to the cardiac toxicity of antineoplastic therapy. It is important to accompany patients undergoing treatment with cardiotoxic potential and monitor them to perform early cardiotoxicity diagnosis and start early treatment (Rocha et al., 2013). However, there is still no conclusive evidence about the benefit of cardioprotective drugs. New research in the area is crucial to develop prevention and treatment strategies (Ribeiro et al., 2019).

As a limitation of the study, the small sample number may have hindered statistical correlation analysis between a cardiotoxicity and risk factors. The use of anthracyclines by patients from both as health units, coding influencing the analysis of results. Studies with more robust given are necessary for extrapolation of the results.

## Conclusion

The results suggest that patients treated at different health units have a high incidence of previous cardiovascular diseases, reinforcing the need for cardiovascular evaluation and monitoring for patients with breast cancer.

Identifying cardiovascular adverse effects allows a better understanding of the toxicity profile of Trastuzumab and the management of adverse effects, thus avoiding treatment interruption.

## Data Availability

The original contributions presented in the study are included in the article/Supplementary Materials, further inquiries can be directed to the corresponding author.
